# Atypical Presentation of a Right Atrial Myxoma

**DOI:** 10.7759/cureus.47084

**Published:** 2023-10-15

**Authors:** Guarina Molina, Rafael Contreras, Melissa Alvarez, Jason Goodman, Arshad Yekta

**Affiliations:** 1 Internal Medicine, Danbury Hospital, Danbury, USA; 2 Cardiology, Danbury Hospital, Danbury, USA; 3 Cardiology, Norwalk Hospital, Norwalk, USA

**Keywords:** pathology slides, surgical case reports, cardiothoracic & vascular surgery, right atrial myxoma, cardiac tumor in adults

## Abstract

Primary cardiac tumors are exceptionally rare and predominantly located in the left atrium with occasional involvement on the right side of the heart. We present the case of a 52-year-old man who presented with chest pain, leading to suspicion of acute coronary syndrome. However, further investigation revealed a right atrial tumor measuring 6.3 cm. After surgical removal, the pathology analysis of the mass confirmed the histology of myxoma. Differential diagnoses for atrial myxomas include thrombus and other tumors, such as rhabdomyomas. More than half of these tumors arise in the left atrium and may be complicated by neurologic symptoms secondary to embolization. Right atrial myxomas are rare and described in the literature with a myriad of symptoms (signs of right heart failure [i.e., fatigue, peripheral edema, hepatomegaly, ascites], a diastolic murmur, and symptoms of pulmonary emboli). In other cases, they may be asymptomatic. Due to the low incidence and variety in their clinical picture, careful documentation of these cases is suggested for early recognition and directed management.

## Introduction

Primary cardiac tumors are exceptionally rare, with an estimated incidence of only 0.02% [1]. Atrial myxomas account for approximately 15-20% of cases, mostly located in the left atrium with occasional involvement on the right side of the heart [1]. Despite their infrequency, myxomas can lead to diverse clinical presentations: incidental findings to significant alterations in cardiac function, arrhythmias, and embolic events. In this report, we highlight the diagnostic challenges and clinical complexity surrounding atrial myxomas, underscoring the need for heightened clinical awareness. Our case serves as a reminder of the importance of early diagnosis and prompt surgical intervention to mitigate potential neurological and cardiovascular complications associated with atrial myxomas.

This article was previously posted to the Europe PMC preprint service on May 04, 2023.

## Case presentation

A 52-year-old male presented to the emergency department with abrupt, persistent, and worsening chest pressure in the left scapular region, radiating to the left chest and rated as 9 out of 10 in intensity. Days prior, he also experienced left hemi-body paresthesia and intermittent headaches, which were minimally relieved by acetaminophen. His medical history included hypertension and a history of treated tuberculosis. 

He presented afebrile and was hemodynamically stable. Cardiac examination revealed a regular rate and rhythm, with 2 out of 6 systolic ejection murmur. Given the acuity and severity of symptoms, the initial differential diagnoses considered were acute coronary syndrome and aortic dissection.

**Figure 1:**
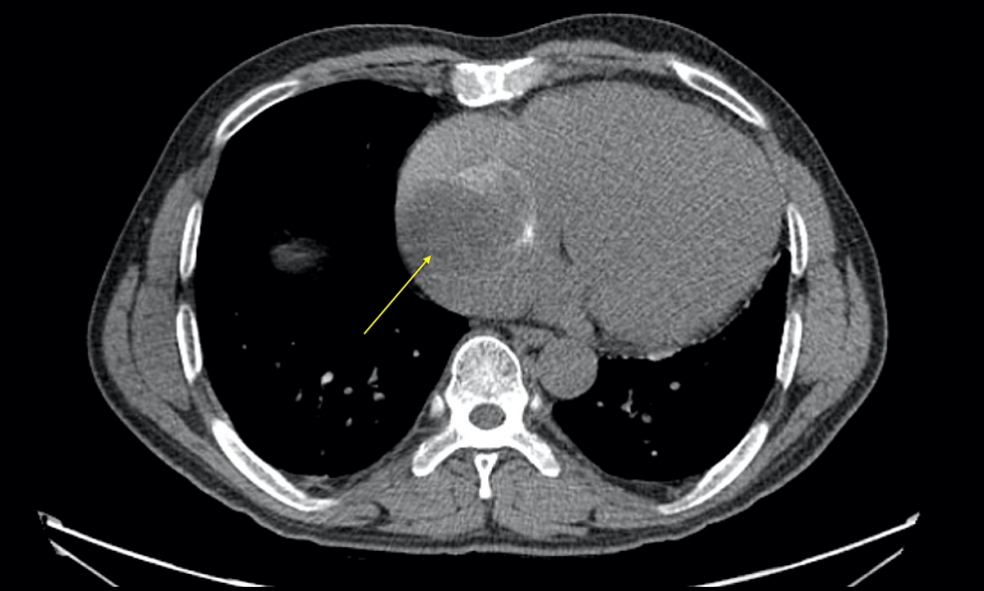
Transverse view of CT of chest Transverse view of CT of chest showing a mass in the cardiac area with calcifications (yellow arrow).

Pertinent bloodwork showed an NT-proBNP level of 1,194 pg/mL and fifth-generation troponin of 10 ng/L, with unremarkable chemistries and blood counts. Further studies included a CT angiogram of the chest, abdomen, and pelvis, which revealed cardiomegaly and a 6.3 cm partially calcified mass in the right atrium, raising suspicion for either atrial myxoma or cardiac thrombus (Figure 1).

**Figure 2:**
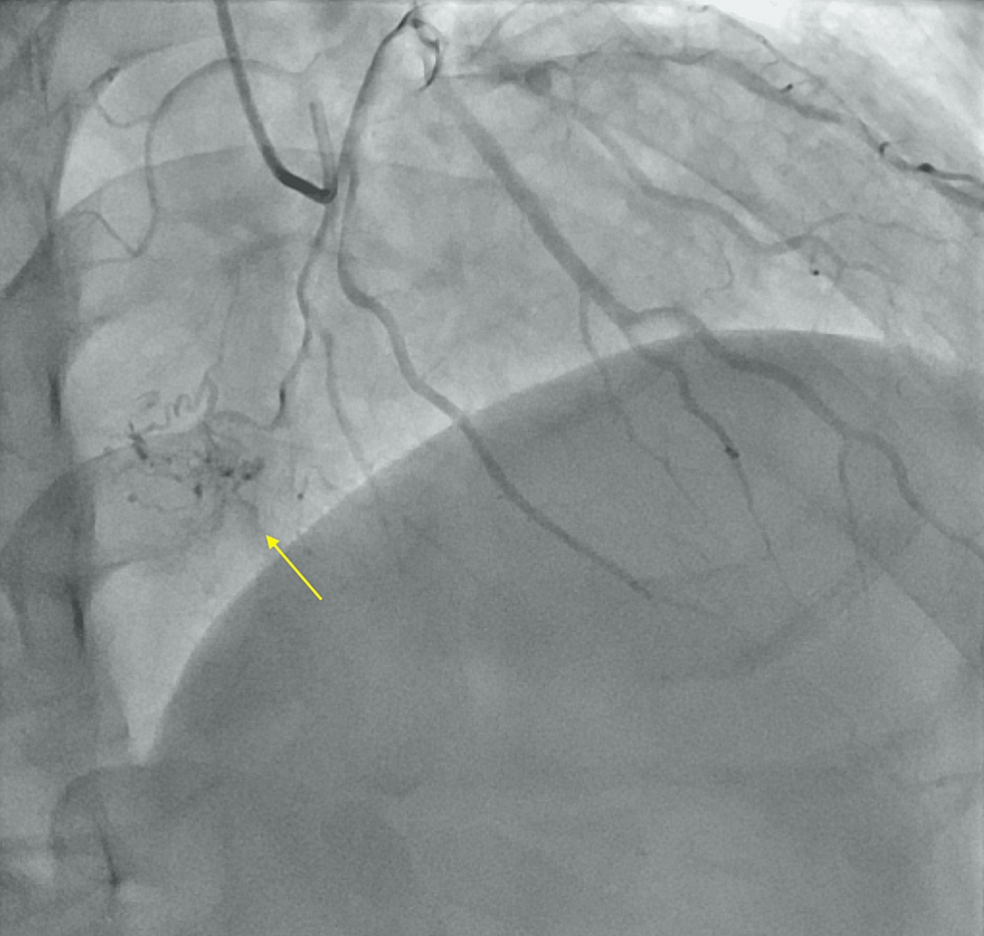
Tumor blush of irrigation of the mass by the LCX (yellow arrow) LCX = left circumflex artery.

A 2D transthoracic echocardiogram demonstrated decreased systolic function with an ejection fraction of 15-20%, along with enlargement of both atria and left ventricle. Left heart catheterization and left and right coronary angiography revealed no vessel occlusion. The left circumflex artery appeared normal in size and appeared to supply blood to the atrial mass (Figure 2). Due to the thrombotic nature of myxomas, a heparin drip was initiated in preparation for surgery.

**Figure 3:**
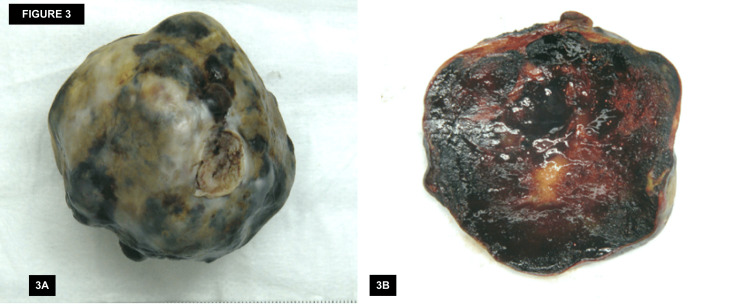
Gross specimen analysis of the resected mass Gross specimen analysis revealed a lobulated, mottled, brown to yellow-red mass of rubbery tissue.

Surgical resection was performed via open heart surgery with identification of a mass of approximately 6.3 x 7 cm with insertion on the interatrial septum. No atrial septal defects were identified. The mass was successfully removed and the created interatrial defect was repaired using a pericardial patch. Pathology of the specimen confirmed an atrial myxoma measuring 7.5 cm × 5.7 cm × 4.6 cm and weighing 88 grams. Gross specimen analysis revealed a lobulated, mottled, brown to yellow-red mass of rubbery tissue (Figure 3A, 3B).

**Figure 4:**
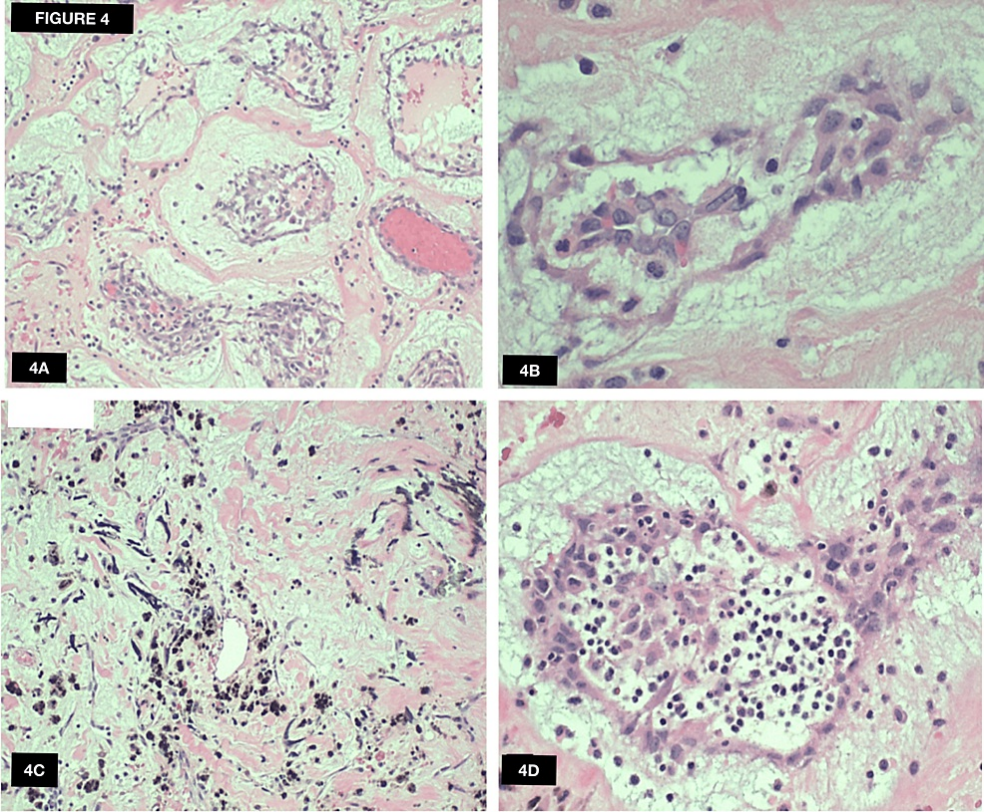
Pathology analysis of the cardiac mass Histological analysis displayed areas of old hemorrhage obscuring neoplastic cells (A, B) and syncytial aggregations of bland pink stellate cells surrounded by a myxoid stromal matrix (C, D).

Histological analysis displayed areas of old hemorrhage obscuring neoplastic cells (Figure 4A, 4B) and syncytial aggregations of bland pink stellate cells surrounded by a myxoid stromal matrix (Figure 4C, 4D).

Postoperatively, subcutaneous heparin was started as deep vein thrombosis (DVT) prophylaxis. Recovery was complicated by generalized tonic-clonic seizures and unresponsiveness, initially attributed to prolonged recovery from anesthesia. Neurologic examination showed right gaze preference, lack of voluntary movements, or withdrawal from noxious stimuli. CT of the brain and head without contrast revealed no evidence of infarction or hemorrhage.

Electroencephalogram displayed abnormal background slowing, suggesting generalized cerebral dysfunction. The patient was started on levetiracetam and minimal sedation, with gradual improvement of mental status.

On the seventh postoperative day (POD), the patient developed a fever of 101.3 F, prompting an infectious workup and a lower extremity (LE) ultrasound to assess for DVT. Shortly after, he complained of left LE pain and imaging confirmed acute DVT involving the left external iliac, common femoral, proximal femoral, deep femoral, and deep popliteal veins. There was no evidence of acute thrombosis in the right limb. Physical assessment reported increased numbness and tenderness of the affected extremity and difficulty moving his toes. Dorsalis pedis and posterior tibialis pulses, which had been palpable, could only be detected with Doppler.

Due to signs concerning compartment syndrome, the patient was started on apixaban, and by POD 9 he was deemed medically stable for discharge. His discharge medications included apixaban 5 mg BID, levetiracetam 1,000 mg BID, metoprolol tartrate 12.5 mg daily, and midodrine 10 mg daily. While guideline-directed medical therapy was required due to his recently discovered non-ischemic cardiomyopathy, it was considered more appropriate to initiate it in the outpatient setting.

At a one-month neurology follow-up, the patient remained compliant with the levetiracetam regimen and denied any recurrence of seizures. He was advised to avoid driving and seizure triggers and a 24-hour electroencephalogram was requested. From a cardiothoracic standpoint, there was adequate recovery with no postoperative complications, leading to the discontinuation of apixaban four months later. Unfortunately, the patient was lost to follow-up with the cardiology service; however, medical records do not indicate any recurrent incidents or hospital admissions at nine months.

## Discussion

Cardiac tumors can present as incidental findings or through alteration of physiological mechanisms: obstruction of cardiac flow, arrhythmias, pericardial effusion, embolization, or constitutional symptoms [1]. Cardiac myxoma is the most common type of primary heart tumor, mostly located in the left atrium and arising from the interatrial septum. The average age at presentation is 50 years, but they usually occur between the third and sixth decades of life [2] with a 3:1 female predominance. Myxomas from the right chambers are rare and represent 15-28% of all cardiac tumors, usually arising from the atrial walls near the fossa ovalis and often with associated calcification [3].

Non-specific symptoms include fever, weight loss, arthralgia, Raynaud phenomenon, and elevation of inflammatory markers due to increased levels of circulating IL-6 [1,4,5]. Right-sided myxomas can also be completely asymptomatic leading to a delayed presentation [5]. Neurologic manifestations such as syncope, stroke, and seizures, although infrequent, have also been described in the literature [6,7]. If they present with angina, they may be mistaken as coronary artery disease (CAD); however, in myxomas, chest pain occurs due to tumor dislodgement and embolization into the pulmonary vasculature [4]. An ischemic workup should still be pursued in these patients, as up to two-thirds of patients with a myxoma can have concomitant CAD. 

Physical examination can be completely non-contributory, while at times, a "tumor plop" may be heard according to the location and size of the mass. Myxomas can be associated with left-sided heart failure (HF), or in rare cases, right-sided HF. In our patient, the cause and timeline of such severe biventricular dilation and low ejection fraction in relationship to mass growth are yet to be investigated.

Surgical removal of symptomatic myxomas is the gold standard of treatment and should be performed as soon as possible after diagnosis [7]. Assurance of safe margins is essential for complete management to prevent recurrence. Foci of calcification that represent areas of necrosis and hemorrhage within the tumor could explain their thrombogenicity [4]. Our patient developed a DVT in the intermediate postoperative period, which could be attributed to prothrombogenic factors arising from the tumor remaining in the bloodstream and subsequent venous stasis. His altered mentation and seizures may be related to the open chamber procedure as well as the use of cardiopulmonary bypass [8], but continued neurologic follow-up has shown no recurrence of seizures at the two-month follow-up.

The American Heart Association reports that hypercoagulable states are common in cardiac surgery, with pro-thrombotic states reaching a peak between days 3 and 5 and can last up to a month. This is suggested to be secondary to increased concentration of clotting factors (fibrinogen, thrombin) and aspirin resistance. Evidence suggests DVT prophylaxis could reduce the risk of pulmonary embolism and symptomatic DVT after cardiac surgery [9].

Cardiac myxomas are rare primary cardiac tumors that may be missed due to the subtle and non-specific nature of their symptoms, leading to delays in diagnosis and management. They may present alone or as part of familial genetic disorders which are out of the scope of this case. Once the diagnosis is made, surgical resection is paramount to reduce the risk of neurologic and cardiovascular complications.

## Conclusions

Cardiac tumors, particularly cardiac myxomas, can manifest as incidental findings or disrupt normal physiological mechanisms. While relatively rare, they represent the most common type of primary heart tumor, with a notable female predominance and typically occurring between the third and sixth decades of life. Accurate diagnosis is crucial, as cardiac myxomas can have serious cardiac and neurological complications. Due to the subtle and non-specific nature of their symptoms, cardiac myxomas may be missed or misdiagnosed, underscoring the need for increased awareness to achieve optimal patient outcomes.
